# Cathepsin B overexpression induces degradation of perilipin 1 to cause lipid metabolism dysfunction in adipocytes

**DOI:** 10.1038/s41598-020-57428-6

**Published:** 2020-01-20

**Authors:** Yuhei Mizunoe, Masaki Kobayashi, Shunsuke Hoshino, Ryoma Tagawa, Rei Itagawa, Ayana Hoshino, Naoyuki Okita, Yuka Sudo, Yoshimi Nakagawa, Hitoshi Shimano, Yoshikazu Higami

**Affiliations:** 1 Department of Internal Medicine (Endocrinology and Metabolism), Faculty of Medicine, University of Tsukuba, Ibaraki, Japan; 2 Laboratory of Molecular Pathology & Metabolic Disease, Faculty of Pharmaceutical Sciences, Tokyo University of Science, Chiba, Japan; 3 Department of Pathological Biochemistry, Faculty of Pharmaceutical Sciences, Yamaguchi Tokyo University of Science, Yamaguchi, Japan; 4 International Institute for Integrative Sleep Medicine (WPI-IIIS), University of Tsukuba, Tsukuba, Japan; 5 Life Science Center for Survival Dynamics, Tsukuba Advanced Research Alliance (TARA), University of Tsukuba, Ibaraki, Japan; 6 AMED-CREST, Japan Agency for Medical Research and Development (AMED), Tokyo, Japan

**Keywords:** Lysosomes, Obesity

## Abstract

Obesity, caused by the dysfunction of white adipose tissue (WAT), is reportedly accompanied by exacerbation of lipolysis. Perilipin 1 (PLIN1), which forms a coat around lipid droplets, interacts with several lipolysis proteins to regulate lipolysis. While it is known that perilipin family proteins are degraded in lysosomes, the underlying molecular mechanisms related to the downregulated expression of PLIN1 in obese WAT remain unknown. Recently, we found that lysosomal dysfunction originating from an abnormality of cathepsin B (CTSB), a lysosomal representative protease, occurs in obese WAT. Therefore, we investigated the effect of CTSB alterations on PLIN1 expression in obese WAT. PLIN1 protein disappeared and CTSB protein appeared in the cytoplasm of adipocytes in the early stage of obese WAT. Overexpression of CTSB reduced PLIN1 protein in 3T3L1 adipocytes, and treatment with a CTSB inhibitor significantly recovered this reduction. In addition, CTSB overexpression induced the dysfunction of lipolysis in 3T3L1 adipocytes. Therefore, we concluded that upregulation of CTSB induced the reduction of PLIN1 protein in obese WAT, resulting in lipolysis dysfunction. This suggests a novel pathology of lipid metabolism involving PLIN1 in adipocytes and that CTSB might be a therapeutic candidate molecule for obese WAT.

## Introduction

White adipose tissue (WAT) is composed of many adipocytes and functions to store surplus fuel as lipid droplets (LDs) and supply it as required, but excess WAT leads to obesity^1–3^. Perilipin coats LDs in adipocytes, thus playing a role in their stabilization and lipolysis by lipases and their cofactors^4^. The perilipin family is composed of five proteins: perilipin 1 (PLIN1), perilipin 2 (adipophilin/PLIN2), perilipin 3 (TIP47/PLIN3), perilipin 4 (S3-12/PLIN4), and perilipin 5 (OXPAT/MLDP/PLIN5). PLIN1 is highly expressed in WAT and is the most studied member of the perilipin family^4–6^. Under lipolytic conditions such as isoproterenol treatment, cAMP-dependent protein kinase phosphorylates hormone-sensitive lipase (HSL) and PLIN1. Phosphorylation of HSL induces its catabolic activity and translocation from the cytosol to LD, while phosphorylation of PLIN1 changes its conformation to facilitate the attachment of activated HSL to LD, leading to the hydrolysis of triacylglycerol to diacylglycerol and fatty acid^7–9^. Notably, the phosphorylation of PLIN1 at serine (Ser)-492 and Ser-517 was associated with basal and hormone-stimulated lipolysis^7–9^. Moreover, mice with a systemic deletion of PLIN1 showed activated basal lipolysis and attenuated stimulated lipolysis. Collectively, these findings indicate that PLIN1 regulates lipolysis and adipose homeostasis^10,11^. In addition, PLIN1 was downregulated in obese WAT, and this resulted in the dysregulation of lipid metabolism including lipolysis^12^. Several studies have addressed the regulation of PLIN1 expression. For example, the reduced expression of PLIN1 protein in obesity was associated with the promotion of inflammatory responses^13^. The *Plin1* gene is epigenetically regulated, and methylation of its promoter was inversely correlated with basal lipolysis in obese women^14^. Despite these findings, to date, no consensus has been reached on the mechanisms underlying the obesity-associated downregulation of PLIN1.

Lysosomes are acidic organelles within cells that contain many digestive hydrolases including lipases, phosphatases, and proteases, which degrade specific substrates^15,16^. Cathepsins are representative lysosomal proteases that play a major role in the degradation of specific proteins^17,18^. Cathepsins are divided into three groups: aspartyl, cysteine, and serine. The aspartyl cathepsins are represented by cathepsin D (CTSD) and cathepsin E, whereas cysteine cathepsins include cathepsin B (CTSB), and cathepsin L (CTSL), CTSB, CTSL and CTSD are the most abundant lysosomal proteases^19^. In addition, these cathepsins are involved in the pathogenesis of cancer, neurodegeneration, and metabolic diseases^15,18,20^. Many studies have reported that the dysfunction of lysosomal cathepsins occur in obese metabolic organs such as WAT and liver, which underlies part of the obesity-related pathology^21^. Recently, we reported that lysosomal alterations in obese WAT impaired autophagic clearance and were involved in the early pathology of obesity^22^. In this previous report, we demonstrated that alterations of CTSL maturation, followed by downregulation of CTSL enzymatic activity during the early development of obesity in WAT exacerbated autophagic clearance leading to autophagosome accumulation^22^. Moreover, complementary activation of CTSB caused by the downregulation of CTSL enhanced inflammasome activation, leading to inflammatory responses in obese WAT^22^. Therefore, it is important to investigate the influence of cathepsin alterations on obese WAT in detail.

Recently, the degradation of perilipins by lysosomal machinery was reported^23,24^, and PLIN2 and PLIN3 were reported as targets for chaperone-mediated autophagy (CMA)^25^. Thus, here we investigated the involvement of lysosomal alterations in the downregulation of PLIN1 in obese WAT.

## Results

### Downregulation of PLIN1 expression in obese WAT

**Figure 1:**
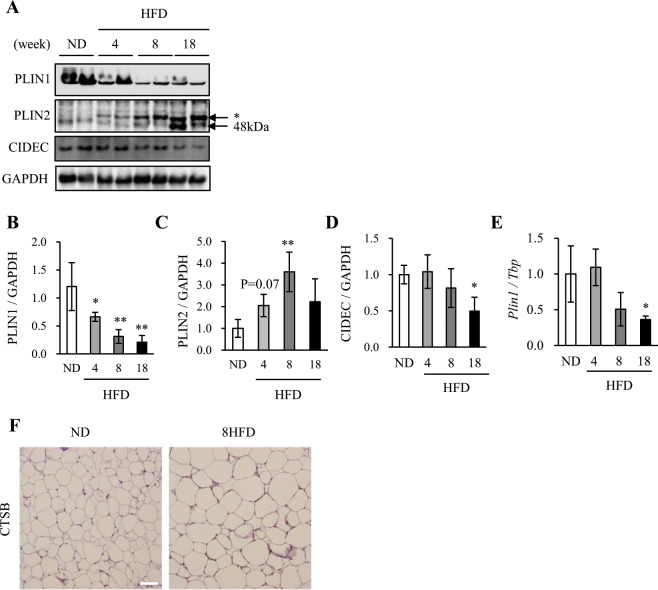
Perilipin 1 (PLIN1) protein levels were downregulated in obese WAT. (**A**) Total protein extracted from WAT of ND, 4HFD, 8HFD and 18HFD mice was analysed by immunoblotting with anti-PLIN1, PLIN2, CIDEC, and GAPDH antibodies (**A**) and quantified (**B**–**D**). Representative images and quantitative data (n = 4) are shown. Intensity of GAPDH was used as a loading control (n = 4). Values indicate mean ± SD. Differences between values were analysed by Student’s *t*-test with Bonferroni correction. *P < 0.05, **P < 0.01. (**E**) mRNA expression levels of *Plin1* in WAT of ND, 4HFD, 8HFD and 18HFD mice, as analysed by real-time RT-PCR (n = 4). Data were normalized against *Tbp* (n = 4). Values indicate the mean ± SD. Differences between values were analysed by Student’s *t-*test with Bonferroni correction. *P < 0.05, **P < 0.01. (**F**) Immunoenzymatic staining of CTSB in WAT of ND and 8HFD mice. Scale bars represent 200 μm.

To confirm the dysregulation of PLIN1 in obese WAT, we compared alterations in the expressions of PLIN1 and PLIN2, a perilipin family that is ubiquitously expressed and which participates in LD formation, accompanying high-fat diet (HFD) feeding over a time-course using the normal diet (ND) group as a control. In the 4HFD, 8HFD and 18HFD (HFD intake for 4, 8 and 18 weeks, respectively) groups, a significant decrease in PLIN1 protein expression was observed (Fig. 1A,B). In contrast, a significant increase in PLIN2 (48 kDa) protein expression was found (Fig. 1A,C). Moreover, expression of cell death inducing DFFA like effector c (CIDEC/Fsp27), a marker of LDs and adipocyte differentiation, was unchanged in the 4HFD and 8HFD groups, but decreased in the 18HFD group (Fig. 1A,D). In contrast to protein levels, *Plin1* mRNA expression was significantly decreased in the 18HFD group, slightly decreased in the 8HFD group, and unchanged in the 4HFD group (Fig. 1E). In addition, the downregulation of PLIN1 in WAT of the 8HFD group was histologically confirmed (Supplementary Fig. 1A). These results suggest that a decrease in PLIN1 protein preceded that of *Plin1* mRNA. Consistent with this finding, immunohistochemical analysis showed increased CTSB protein expression in the WAT of 8HFD mice (Fig. 1F).

### Downregulation of PLIN1 is associated with the upregulation of CTSB expression in obese WAT

**Figure 2:**
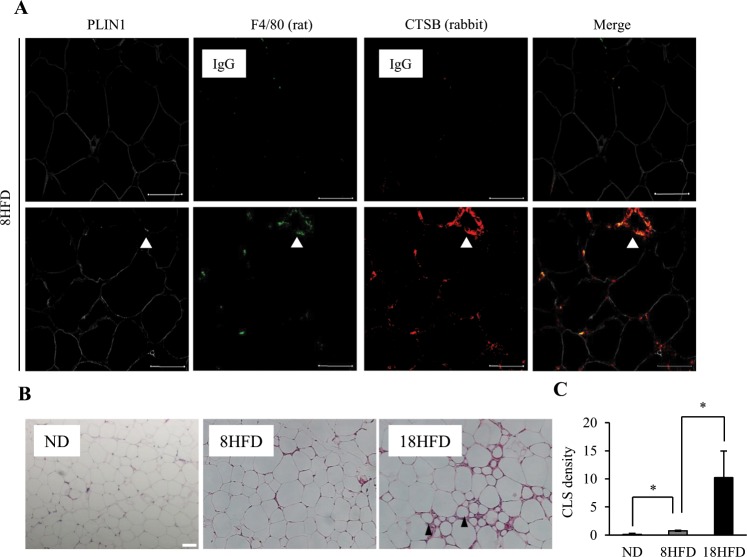
Localization of CTSB between adipocytes and macrophages in obese WAT. (**A**) Representative images of WAT sections from 8HFD mice immunostained for PLIN1, CTSB, and F4/80 (lower panel). Representative images of WAT sections from 8HFD mice immunostained for PLIN1, rabbit-IgG, and rat-IgG (upper panel). Scale bars represent 50 μm. Arrowheads indicate CLS. (**B**,**C**) Increased crown-like structures in 18HFD mice. Representative images (**B**) and quantitative data (CLS per > 300 adipocytes) (**C**) of WAT sections in ND, 8HFD, and 18HFD mice. Arrowheads indicate CLS. Values indicate the mean ± SD. Differences between values were analysed by Student’s *t*-test with *P < 0.05, **P < 0.01.

**Figure 3:**
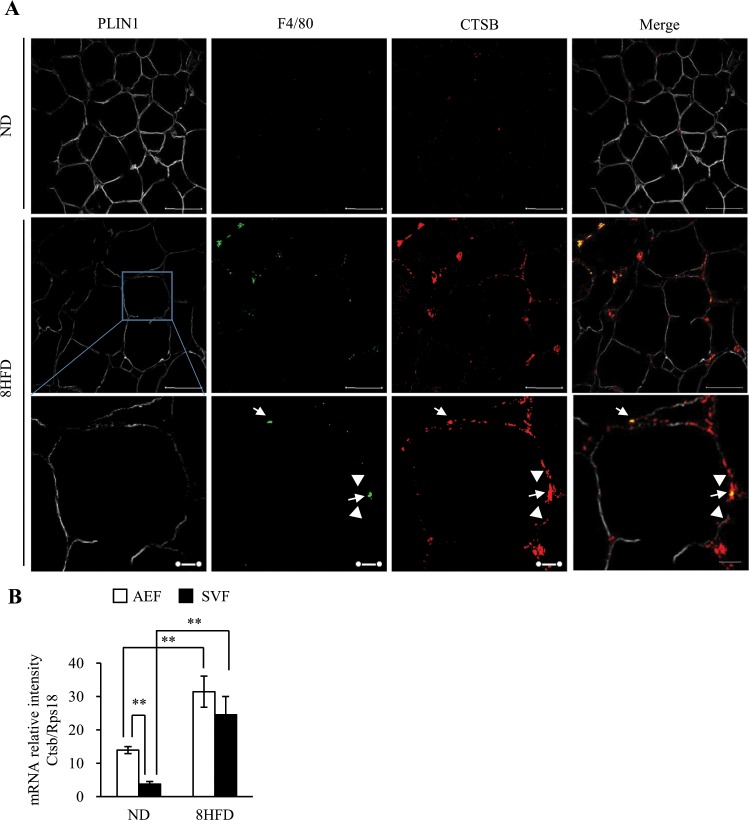
Localization of CTSB in the adipocyte fraction of obese WAT. (**A**) Representative images of WAT sections from ND and 8HFD mice immunostained for PLIN1, CTSB and F4/80. Scale bars represent 50 μm (upper and middle panels) and 10 μm (lower panels). Arrow indicates the co-localization between CTSB and F4/80, and arrowheads indicate the co-localization between CTSB and adipocytes. (**B**) qRT-PCR analysis for *Ctsb* mRNA levels in the adipocyte enriched fraction (AEF) and stromal vascular fraction (SVF) obtained from the WAT of ND and 8HFD groups. Values indicate the mean ± SD. Differences between values were analysed by Tukey-Kramer method with *P < 0.05, **P < 0.01 (n = 4).

We recently reported that CTSB protein was significantly increased in 4HFD, 8HFD and 18HFD mice^22^. These findings suggest that the decrease in PLIN1 protein in early obesity might be associated with an increase in CTSB protein. Generally, it has been reported that macrophage numbers are increased in adipose tissue during obesity and constitute up to 50% of all adipose tissue cells during obesity^1–3^. Therefore, the increased CTSB in obese adipose tissue may be derived from either adipocytes or macrophages. Indeed, we found that CTSB significantly was expressed in macrophages surrounding dead cells, forming so-called “crown-like structures (CLS)” (Fig. 2A). Thus, macrophages that formed CLS showed co-localization with CTSB (Fig. 2A), and CLS was significantly increased in 18HFD mice compared with 8HFD mice (Fig. 2B,C). Taken together, it is likely that the decrease in PLIN1 expression occurs prior to the increase in CLS formation. To clarify whether CTSB localizes in adipocytes or macrophages, we investigated the localization of CTSB protein in ND and 8HFD mice by immunohistochemistry analysis of WAT sections using anti-CTSB and anti-PLIN1 antibodies combined with anti-F4/80 antibody as a marker for macrophages. In agreement with Fig. 1A and previous our work^22^, a reduction in PLIN1 and an increase in CTSB were observed (Fig. 3A). Simultaneously, part of the CTSB positive area was merged with F4/80, suggesting that CTSB protein was localized to macrophages and adipocytes (Fig. 3A, arrow). In ND mice, the expression of *Ctsb* mRNA was significantly higher in the adipocyte enrichment fraction (AEF) than the stromal vascular fraction (SVF), and *Ctsb* expression was increased in both fractions of 8HFD mice (Fig. 3B). Interestingly, we also clearly detected that PLIN1 protein and CTSB protein were alternatively localized in the cytoplasm of adipocytes in the obese WAT of 8HFD mice (Fig. 3A, arrowhead). Therefore, we hypothesized that PLIN1 protein in obese WAT is degraded by increased CTSB in adipocytes at the early stage of obesity, and that increased PLIN1 protein degradation and reduced PLIN1 gene expression contribute to the net protein loss in the late stage of obesity.

### CTSB overexpression induces PLIN1 downregulation in 3T3L1 adipocytes

**Figure 4:**
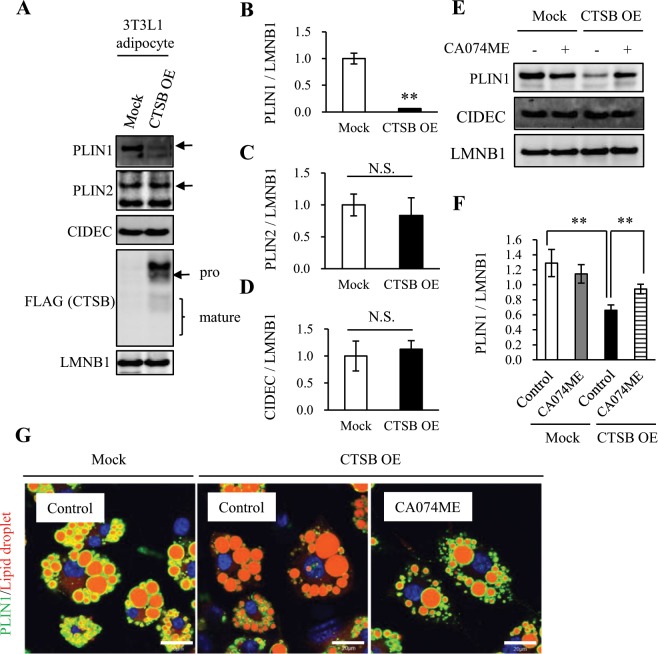
Degradation of PLIN1 by CTSB. (**A**–**D**) Total cell lysates extracted from Mock or CTSB-OE 3T3L1 adipocytes were analysed by immunoblotting using anti-PLIN1, PLIN2, CIDEC, FLAG, and LMNB1 antibodies (**A**) and quantified (**B**–**D**). Representative images and quantitative data (n = 4) are shown. Intensity of LMNB1 was used as a loading control. Values indicate the mean ± SD. Differences between values were analysed by Student’s *t*-test with *P < 0.05, **P < 0.01. (**E**,**F**) Mock or CTSB-OE 3T3L1 adipocytes were treated with 10 μM CA074ME for 24 h. Total cell lysates were analysed by immunoblotting using anti-PLIN1, CIDEC, and LMNB1 antibodies (**E**) and quantified (**F**). Representative images and quantitative data (n = 4) are shown. Intensity of LMNB1 was used as a loading control. Values indicate the mean ± SD. Differences between values were analysed by Tukey-Kramer method with *P < 0.05, **P < 0.01. (**G**) Mock or CTSB-OE 3T3L1 adipocytes were treated with 10 μM CA074ME for 24 h. Immunofluorescence analysis was performed with an anti-PLIN1 antibody. PLIN1 (green), lipid droplets (red) and nucleus (blue) are shown. Data shown are representative of individual experiments. Scale bars represent 20 μm.

To analyse the relationship between PLIN1 expression and CTSB activity, we generated CTSB-OE 3T3L1 adipocytes. A significant decrease in PLIN1 protein was observed in CTSB-OE cells (Fig. 4A,B). In contrast, PLIN2 and CIDEC were unaltered in CTSB-OE cells (Fig. 4A,C,D). Moreover, treatment with CA074ME, a CTSB inhibitor, whose concentration had no effect on cell viability (Supplementary Fig. 2A)^26,27^, rescued PLIN1 expression in CTSB-OE cells (Fig. 4E,F). E64d, another cysteine cathepsin inhibitor analogous to CA074ME, also abrogated the CTSB OE-induced reduction in PLIN1 expression, while pepstatin A, an aspartyl cathepsin inhibitor that suppresses the activity of CTSD and CTSE, failed to exert a similar effect (Supplementary Fig. 2B). However, the mRNA expression of *Plin1* and *Plin2* did not change in CTSB-OE cells with or without CA074ME (Supplementary Fig. 2C,D). These results indicate that CTSB may be associated with reduced PLIN1 protein expression. Immunofluorescent analysis showed that CTSB overexpression further reduced the PLIN1 signal around LDs (Fig. 4G). Similar to the results shown in Fig. 4E,F, this reduction was reversed by treatment with CA074ME (Fig. 4G). These data indicate that CTSB overexpression induced the elimination of PLIN1 protein.

Because PLIN1 protein expression is regulated by peroxisome proliferator activated receptor γ (PPARG), a master regulator of adipogenesis^28^, we considered that the reduced PLIN1 observed in CTSB-OE cells might be attributed to the dysregulation of adipogenesis. To rule out this possibility, we investigated the degree of adipogenesis in CTSB-OE cells and found that triacylglycerol accumulation was significantly increased in CTSB-OE cells (Supplementary Fig. 3A,B). In addition, the expressions of PPARG1 and PPARG2 proteins were more robust over days 2 to 4 during adipocyte differentiation in CTSB-OE cells than in control cells (Supplementary Fig. 3C–E). Moreover, although the expression of CIDEC was unchanged, PLIN1 protein was significantly decreased during adipocyte differentiation in CTSB-OE cells (Supplementary Fig. 3C,F,G). These results indicate accelerated adipogenesis in CTSB-OE cells, in agreement with a previous report^29^. Thus, we concluded that reduced PLIN1 protein in CTSB-OE cells occurs independent of changes in adipogenesis.

### Localization of CTSB and PLIN1 in 3T3L1 adipocytes

**Figure 5:**
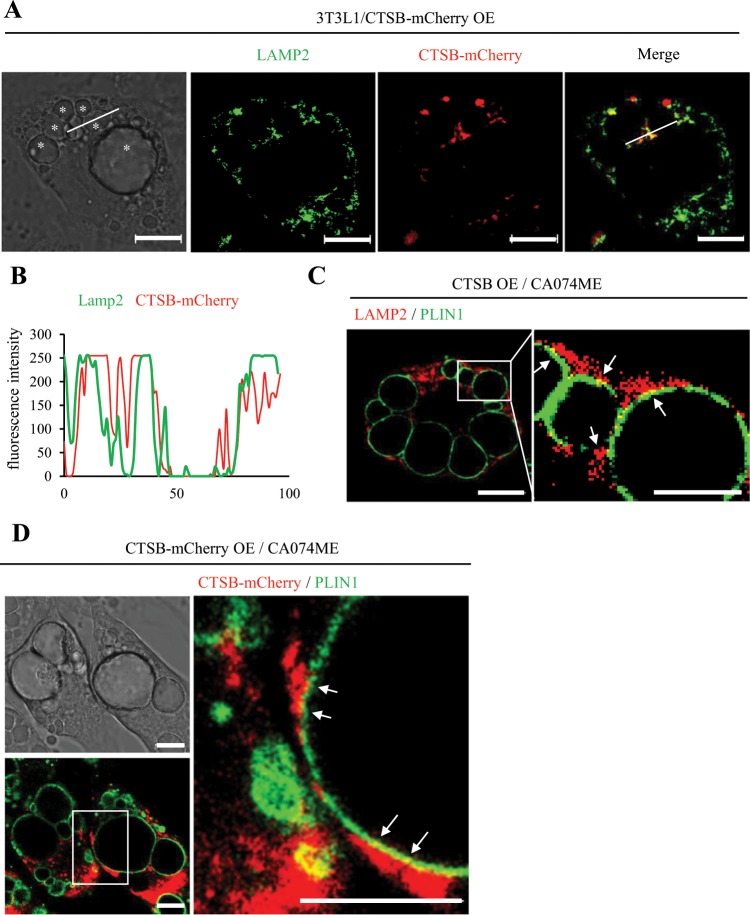
Localization of PLIN1 in CTSB-OE 3T3L1 adipocytes. (A and B) Immunofluorescence analysis was performed with anti-LAMP2 antibodies in CTSB-mCherry-OE 3T3L1 adipocytes. Images of CTSB-mCherry (red) and LAMP2 (green) (**A**), and histogram of fluorescence intensity (**B**) are shown. Representative images of individual experiments are shown. Scale bar represents 10 μm. Asterisk indicates LD. (**C**) CTSB-OE 3T3L1 adipocytes were treated with 10 μM CA074ME for 24 h. Immunofluorescence analysis was performed with anti-PLIN1 and LAMP2 antibodies. PLIN1 (green) and LAMP2 (red) are shown. Representative images of individual experiments are shown. Scale bar represents 5 μm. Arrows indicate the contact site between PLIN1 and LAMP2. (**D**) CTSB-mCherry-OE 3T3L1 adipocytes were treated with 10 μM CA074ME for 24 h. Immunofluorescence analysis was performed with an anti-PLIN1 antibody. PLIN1 (green) and CTSB-mCherry (red) are shown. Representative images of individual experiments are shown. Scale bar represents 5 μm. Arrows indicate the contact site between PLIN1 and CTSB-mCherry.

To determine the localization of CTSB protein in adipocytes, we generated 3T3L1 cells expressing CTSB-mCherry fusion protein, because commercially available CTSB antibodies failed to work for cellular immunostaining in our experiments. In the differentiated CTSB-mCherry-OE adipocytes, most exogenous mCherry was colocalized with lysosomal membrane protein LAMP2, indicating the lysosomal localization of CTSB protein in adipocytes (Fig. 5A,B). Next, to investigate the association between lysosomes and PLIN1, we analysed the localization of LAMP2 and PLIN1 in differentiated 3T3-L1 cells treated with a CTSB inhibitor to facilitate the detection of PLIN1. LAMP2 was in contact with the PLIN1 protein, suggesting that the lysosome interacts with PLIN1 on LDs (Fig. 5C). Furthermore, we identified that CTSB-mCherry was also in contact with PLIN1 (Fig. 5D). These results suggest that PLIN1 protein was associated with CTSB on LDs.

### CTSB overexpression disturbs lipid metabolism

**Figure 6:**
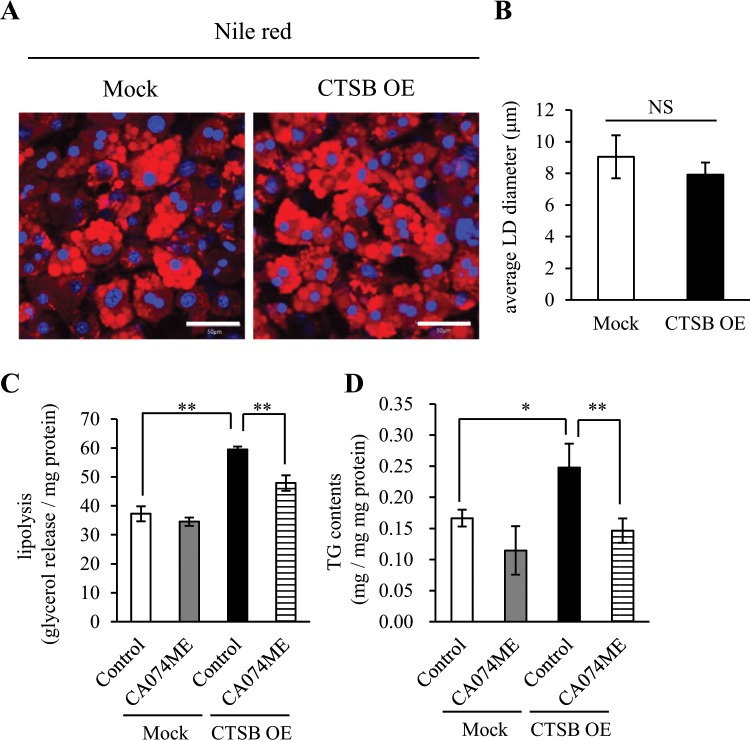
Dysregulation of lipid metabolism by CTSB overexpression in 3T3L1 adipocytes. (A and B) Lipid droplets (LDs) were stained by Nile red in Mock or CTSB-OE 3T3L1 adipocytes. Representative images (**A**) and quantitative data of the mean LD diameter (**B**) are shown. Scale bar represents 50 μm. Values indicate the mean ± SD. Differences between values were analysed by Student’s *t*-test with *P < 0.05, **P < 0.01. (C and D) Release assays for glycerol (**C**) and measurement of intracellular TG content (**D**) were performed in Mock or CTSB-OE 3T3L1 adipocytes. Values indicate the mean ± SD. Differences between values were analysed by Tukey-Kramer method with *P < 0.05, **P < 0.01.

To evaluate the implications of CTSB overexpression-induced PLIN1 downregulation on basal lipolysis, we examined lipolysis, which was evaluated by the release of glycerol into the medium, and intracellular triacylglycerol (TG) content in CTSB-OE cells with or without CA074ME treatment. Initially, we investigated the size of LD in Mock cells and CTEB-OE cells, but failed to detect any differences (Fig. 6A,B). However, CTSB overexpression enhanced the release of glycerol, which was rescued by CA074ME treatment (Fig. 6C). These results indicated that CTSB overexpression induced futile basal lipolysis because of the PLIN1 degradation. Interestingly, CTSB-OE cells exhibited increased intracellular TG content compared with Mock cells, which was inhibited by CA074ME treatment (Fig. 6D). Such phenotypic changes in TG content were associated with increased lipid metabolism^30,31^. Therefore, CTSB overexpression is associated with the alteration of lipid metabolism in adipocytes.

## Discussion

Several studies have demonstrated that obesity reduces PLIN1 expression. For example, Gaidhu *et al*. found that downregulated PLIN1 protein expression in obese WAT caused lipolytic dysfunction in obese WAT^12^. However, the underlying mechanism remains incompletely understood. It was reported that *PLIN1* mRNA expression was lower in WAT from obese individuals compared with that from non-obese subjects^32^. Conversely, recent studies have demonstrated that PLIN1 protein is degraded by lysosomes^23,24,33^. In addition, Kovsan *et al*. showed significantly increased PLIN1 protein expression in adipocytes treated with the lysosomal protease inhibitor leupeptin, but not the lysosomal acidity inhibitor NH_4_Cl, suggesting that PLIN1 protein may be degraded specifically by cathepsin^23^. Leupeptin inhibits sulfhydryl proteases such as plasmin, papain, trypsin, and CTSB^34,35^. Therefore, we hypothesized that CTSB contributes to the degradation of PLIN1 protein in obese WAT. In the present study, we demonstrated that CTSB overexpression led to reduced PLIN1 protein and altered lipid metabolism in 3T3L1 adipocytes. Furthermore, a decrease in PLIN1 protein levels occurred in the early stage of HFD (4HFD), but *Plin1* mRNA levels failed to significantly decrease until the late stage of HFD (18HFD). Therefore, we speculated the decrease of PLIN1 protein in 4HFD and 8HFD may be mainly related to the upregulation of CTSB activity, whereas in 18HFD, the downregulation of *Plin1* mRNA levels might synergistically contribute to decreased PLIN1 protein in addition to protein degradation by CTSB. Considering a previous study reporting that the *Plin1* gene is epigenetically regulated^14^, we speculated that *Plin1* mRNA changes might be related to epigenetic regulation in the late stage of obesity. Furthermore, we observed that PLIN2 protein abundance was increased in obese WAT unlike PLIN1. It was reported that PLIN2 proteins accumulated in CLS macrophages in obese WAT^36^. Therefore, given our result that PLIN2 protein is unchanged in CTSB-OE cells, it is likely that the obesity-induced upregulation of PLIN2 in WAT may be attributed to the increased infiltration of macrophages, rather than CTSB overexpression in obese adipocytes.

In addition to CTSB, we also identified that CTSD, a lysosomal serine protease ubiquitously expressed in various cells/tissues, was upregulated in obese WAT (Supplementary Fig. 4A). Therefore, to clarify the relationship between CTSD and PLIN1, we used CTSD-OE 3T3L1 adipocytes and examined the effects on PLIN1 expression (Supplementary Fig. 4B). CTSD overexpression, however, did not affect PLIN1 protein levels (Supplementary Fig. 4C–E). These results suggest that CTSD, a major lysosomal hydrolase, does not contribute to the degradation of PLIN1. It was reported that CTSB directly degraded tenascin-C, an extracellular matrix glycoprotein^37,38^. We showed here that CTSB was involved in the reduction of PLIN1 in obese WAT. Therefore, it is likely that PLIN1 is a novel-selective substrate of CTSB.

Regarding how CTSB and PLIN interact with each other, we revealed that exogenous CTSB protein was mainly localized in lysosomes in CTSB-OE cells and in contact with PLIN1 protein on LDs (Fig. 5). We propose two mechanisms for this phenomenon as follows. One possibility involves chaperone-mediated autophagy (CMA), which selectively degrades a subset of cytosolic proteins by direct sequestering in lysosomes. As stated above, both PLIN2 and PLIN3 proteins are targets of CMA^25^. Because the perilipin family shares 43% amino acid sequence identity^4^, PLIN1 protein might also be a target of CMA. A second possibility is that CTSB released by lysosomal membrane permeabilization (LMP) interacts with PLIN1 in cytoplasm^39,40^. Several factors, such as oxidative stress, fatty acids, and lysosomotropic compounds, induce LMP and cause the release of cathepsins and other hydrolases from the lysosomal lumen into the cytosol^39^. This release occurs under conditions of cell death including apoptosis or necrosis^39^. In obese WAT, PLIN1 protein is not detected by immunohistochemistry during adipocyte cell death, which is generally characterized by increased CLS^36^. These reports support the notion that CTSB released by LMP causes the degradation of the PLIN1 protein in obese WAT. However, our results also indicate that decreased PLIN1 expression occurs prior to the increase in CLS (Figs. 1–3). Thus, we suggest that CMA and the release of CTSB accompanying LMP might play a role in PLIN1 degradation at the early and advanced stages of obesity, respectively. In the future, it will be important to examine further the involvement of CMA and LMP in the degradation of PLIN1.

PLIN1 has multiple important phosphorylation sites (Ser-434, Ser-492, and Ser-517) in its C-terminal region that regulate its function^4^. It is generally accepted that lipolysis is dysregulated in the WAT of obese mice and obese humans, attributable to decreased PLIN1 and HSL protein expressions^12,32,41,42^. Moreover, in PLIN1-knockout mice, basal lipolysis was induced, but stimulated lipolytic activity was attenuated^11^. PLIN1 knockdown in 3T3-L1 adipocytes dramatically increased basal lipolysis^43^. Our results also showed that the CTSB-induced degradation of PLIN1 promoted basal lipolysis. These findings imply that CTSB overexpression causes dysfunctional basal lipolysis through PLIN1 downregulation in obese WAT. However, CTSB-OE cells displayed accelerated adipocyte differentiation and increased TG content, the latter of which was suppressed by CA074ME, implying that CTSB may directly regulate lipogenesis separate from adipocyte differentiation. A previous study showed that lipolysis and lipogenesis were regulated in parallel in adipocytes during the remodelling of LDs^30,31^, which supports the idea that increased TG levels in CTSB-OE cells might occur independently of alterations in lipolysis. Therefore, although hypertrophic adipocytes with increased TG content were suspected to release more glycerol, we assumed that CTSB overexpression contributed to increased basal lipolysis via reduced PLIN1 and lipogenesis via unknown mechanisms. It is widely accepted that FFAs released from hypertrophic adipocytes activate macrophages primarily via the Toll-like receptor 4-JNK pathway and induce proinflammatory signals^13,44,45^. Therefore, the upregulation of basal FFA released via CTSB-induced PLIN1 degradation in adipocytes might contribute to a chronic state of low-grade inflammation, which is usually observed in obese WAT, leading to a pathological state of obesity.

In conclusion, our data provide the first evidence that CTSB overexpression in obese WAT is associated with reduced PLIN1 protein expression, resulting in abnormal futile basal lipolysis and a possible subsequent inflammatory response. This study proposes an important mechanism of PLIN1 regulation and the possibility that CTSB might be a novel therapeutic target for obesity.

## Materials and Methods

### Animals and diets

All animal experiments and protocols were conducted in accordance with the Fundamental Guidelines for Proper Conduct of Animal Experiment and Related Activities in Academic Research Institutions under the jurisdiction of the Ministry of Education, Culture, Sports, Science and Technology of Japan, and were approved by the Ethics Review Committee for Animal Experimentation at the Tokyo University of Science (approval number Y14028). Male C57BL/6 mice (3 weeks of age) were purchased from CREA Japan (Tokyo, Japan) and maintained in temperature-controlled, specific pathogen-free conditions with 12-h light/dark cycles within the animal facility at Tokyo University of Science. For experiments, male C57BL/6 mice (4 weeks of age) were randomly divided into four groups: normal diet (ND; Nosan, Yokohama, Japan), 4HFD, 8HFD and 18HFD. The latter three groups were fed a high-fat diet (HFD32, CREA) for 4, 8 or 18 weeks, respectively, before euthanasia, whereas ND mice were fed a ND throughout their lives. After 22 weeks, all four groups of mice were euthanized and epididymal WAT was collected.

### Cell culture and treatment

3T3-L1 pre-adipocytes were purchased from RIKEN Bioresource Center (Ibaraki, Japan) and maintained in Dulbecco’s Modified Eagle’s Medium (low glucose; 041–29775, Wako, Osaka, Japan) with 10% foetal bovine serum and 1% penicillin/streptomycin (Sigma-Aldrich, St. Louis, MO, USA). Differentiation of 3T3-L1 pre-adipocytes to adipocytes (Day 8) was performed as previously reported by our laboratory^46,47^. Pepstatin A1 (4397-v) and CA074ME (4323-v) were purchased from Peptide Institute (Osaka, Japan).

### Retrovirus vector construction and infection of 3T3L1 pre-adipocytes

Retrovirus-CTSB overexpression vector and retrovirus-CTSB-mCherry were constructed according to a previous report^22^. Briefly, CTSB cDNA was amplified from a CTSB vector (11249, Addgene, Cambridge, MA, USA) by PCR using PrimeSTAR HS DNA polymerase (R010A, Takara, Kyoto, Japan), and then subcloned into a *Xho*1- and *Not*1-digested pMXs-AMNN-Puro vector^46,47^. Oligonucleotide primers for CTSB were chemically synthesized. CTSD cDNA was amplified from mouse cDNA libraries using the chemically synthesized primers. For the CTSB-mCherry vector, CTSB cDNA was subcloned into a Xho1- and HindIII-digested pmCherry-N1 vector. Subcloned vectors were then digested with Xho1 and Not1 and cloned into a Xho1- and Not1-digested pMXs-AMNN-Puro vector^46,47^. The sequences of used primers are shown in the primer list (Supplementary Fig. 5A). Retroviral vectors were produced by transfecting Plat-E cells with the resulting vector plasmids, as described previously^48^. The retrovirus particles were concentrated with a 4 × PEG-it solution (32% (*w/v*) PEG-6000, 400 mM NaCl, and 40 mM HEPES, pH 7.4). To obtain 3T3L1 cell lines overexpressing CTSB, CTSD, or CTSB-mCherry, 3T3L1 cells were incubated with virus-containing medium for 2 days and then selected with 2 μg/mL puromycin for 5 days.

### Immunoblotting analysis

Immunoblotting with Immunostar LD chemiluminescent substrates (290–69904, Wako) was performed. Signals were detected with an LAS-3000 image analyser (Fujifilm, Tokyo, Japan) as previously described^49,50^. Lamin β1 (LMNB1; PM064, 1:10000) was purchased from MBL (Nagoya, Japan). CTSB (ab58802, 1:400), CIDEC/FSP27 (ab16760, 1:2000), PLIN1 (ab61682, 1:400) and PLIN2 (ab108323, 1:1000) were purchased from Abcam (Cambridge, UK). FLAG M2 (F1804, 1:5000) was purchased from Sigma-Aldrich. PPARG (sc-7273, 1:2000) was purchased from Santa Cruz Biotechnology (Santa Cruz, CA, USA). GAPDH (glyceraldehyde-3-phosphate dehydrogenase) (010-25521, 1:5000) was purchased from Wako.

### Cathepsin activity assay

Cathepsin activity was assayed as previously reported^22,51^. Briefly, fluorometric analysis with Z-Arg-Arg-MCA (3123-v, Peptide Institute) for CTSB and MOCAc-Gly-Lys-Pro-Ile-Leu-Phe-Phe-Arg-Leu-Lys(Dnp)-D-Arg-NH_2_ for CTSD (3200-v, Peptide Institute) was performed as previously reported^22^ using WAT or cell pellets resuspended in lysis buffer (352 mM KH_2_PO_4_, 48 mM Na_2_HPO_4_, 4 mM EDTA, pH 6.0) and incubated on ice for 60 min before centrifugation for 10 min at 2100 × g. Supernatant was collected and protein concentrations were determined with a BCA kit (23225, Pierce, Waltham, MA, USA). Supernatant was then added to the reaction buffer (4 mM DTT in lysis buffer) as an assay buffer.

For the measurement of CTSB activity, 100 μL assay buffer (containing 1 μg of protein) was mixed with 100 μL of substrate buffer (10 μM Z-Arg-Arg-AMC diluted in 0.1% Brij 35; B4184, Sigma-Aldrich) and incubated at 37 °C for 30 min. Fluorescence was measured using a Wallac ARVO MX/Light 1420 Multilabel/Luminescence Counter (PerkinElmer, Waltham, MA, USA) with an excitation/emission of 360/460 nm.

For the measurement of CTSD activity, 100 μL of assay buffer (containing 1 μg of protein) was mixed with 100 μL of substrate buffer (10 μM CTSD substrate diluted in 0.1% Brij 35) and incubated at 37 °C for 30 min. Fluorescence was measured using a Wallac ARVO MX/Light 1420 Multilabel/Luminescence Counter with an excitation/emission of 360/460 nm.

### Quantitative real-time RT-PCR

Total RNA was extracted from frozen WAT using the ReliaPrep RNA Tissue Miniprep System (Promega, Tokyo, Japan), in accordance with the manufacturer’s protocol. To obtain cDNA, 1 μg of RNA was subjected to reverse transcription using PrimeScript Reverse Transcriptase (Takara, Shiga, Japan) and random hexamer primers (Takara). Quantitative real-time RT-PCR was performed using a CFX Connect Real-Time PCR System (Bio-Rad, Hercules, CA, USA) with Thunder Bird SYBR qPCR Mix (QPS-201, Osaka, Japan), as previously described^52^. The sequences of used primers are shown in the primer list (Supplementary Fig. 5B).

### Immunofluorescence and confocal microscopy in 3T3L1 adipocytes

3T3L1/Mock, 3T3L1/CTSB-overexpressing (OE), and 3T3L1/CTSB-mCherry-OE pre-adipocytes grown on cover slips were differentiated into adipocytes and treated with or without 10 μM of the cathepsin B inhibitor CA074ME (4323-v, Peptide Institute) for 24 h. After fixing with 4% paraformaldehyde for 15 min, cells were permeabilized and incubated with primary antibodies against PLIN1 (ab61682, 1:400, Abcam) or LAMP2 (ab13524, 1:1000, Abcam) at 4 °C overnight, and then with Alexa Fluor-conjugated secondary antibodies (Alexa Fluor-488 goat anti-rabbit IgG (H + L), A11070, 1:1000, Invitrogen, Carlsbad, CA, USA; Alexa Fluor-594 goat anti-rabbit IgG (H + L), A110121, Invitrogen; Alexa Fluor-488 goat anti-rat IgG (H + L), ab150157, Abcam) for 1 h at room temperature. Hoechst 33342 (H3570, 1:10000, Invitrogen) and Nile red were used to stain nuclei and LDs, respectively. Images were acquired on an SP8 confocal microscope (Leica, Wetzlar, Germany) at 63 × magnification.

### Immunohistochemistry of WAT

WAT samples from ND and 8HFD mice were fixed with 10% formalin for 24 h and embedded in paraffin. Sections (5 μm thick) were cut from each tissue block, deparaffinized, and dehydrated. Antigen retrieval was performed on samples by heating in 10 mM citrate buffer solution (pH 6.0) supplemented with 0.1% NP40 in an autoclave for 10 min at 121 °C. After blocking of nonspecific reactivity with bovine serum albumin and goat serum for 60 min at room temperature, sections were incubated overnight at 4 °C with anti-PLIN1 (GP29, 1:200, Progen Biotechnik, Heidelberg, Germany), anti-F4/80 (ab6640, 1:200, Abcam) and anti-CTSB (#31718, 1:200, Cell Signaling Technology, MA, USA) antibodies. As a negative control, sections were incubated with normal rabbit IgG (sc-2027, Santa Cruz Biotechnology) or normal rat IgG (sc-2026, Santa Cruz Biotechnology). Distribution of the primary antibody was achieved by subsequent application of Alexa Fluor-conjugated secondary antibodies (Alexa Fluor-488 goat anti-rat IgG (H + L), A-11006, 1:1000, Invitrogen; Alexa Fluor-555 goat anti-rabbit IgG (H + L), A27039, Invitrogen; Alexa Fluor-405 goat anti-guinea pig IgG (H + L), ab175678, Abcam) for 1 h at room temperature. Representative images were acquired with a confocal microscope (SP8, Leica) at 63 × oil magnification.

Immunohistochemical analysis of CTSB protein expression was performed following the manufacturer’s protocol for the Histofine SAB-PO kit (424032, Nichirei Bioscience, Tokyo, Japan). Briefly, antigen retrieval was performed on samples as described above. Next, endogenous peroxidase was blocked by incubating sections in 0.3% H_2_O_2_ in dH_2_O for 30 min. After blocking of nonspecific reactivity with goat serum for 60 min at room temperature, sections were incubated overnight at 4 °C with an anti-CTSB (sc-13985, 1:5000, Santa Cruz Biotechnology) antibody. As a negative control, sections were incubated with rabbit IgG (2729, Cell Signaling Technology). Distribution of the primary antibody was achieved by subsequent application of a biotinylated anti-rabbit IgG antibody for 1 h at room temperature, followed by incubation for 5 min with peroxidase-labelled streptavidin. Immunostaining was developed using DAB solution (425011, Histofine DAB Substrate Kit, Nichirei Bioscience). Representative images were acquired with a fluorescence microscope (DMi8, Leica) at 20 × magnification.

### Light microscopy and morphometry

WAT sections (5 μm thick) were stained with haematoxylin and eosin to assess morphology. Images were acquired with a light microscope (Eclipse 80i; Nikon, Tokyo, Japan) using a 10 × objective lens. CLS density (CLS per more than 300 adipocytes) was quantified by ImageJ software (https://imagej.nih.gov/ij/).

### Glycerol release assay and intracellular TG content

A glycerol release assay (Adipolysis assay kit; Cayman Chemical, Ann Arbor, MI, USA) with medium from differentiated 3T3-L1/Mock and 3T3L1/CTSB-OE adipocytes and intracellular TG content (LabAssay™ Triglyceride, Wako) was performed according to the manufacturer’s protocols. Glycerol content in the media was measured calorimetrically using a set of standards. Cells were then washed with cold PBS, lysed in 2% SDS buffer, and the protein concentration was determined and used to normalize glycerol release. All experiments were carried out in triplicate.

### Statistical analyses

Statistical analyses were performed using the Tukey–Kramer test (with R software version 3.1.0) or Student’s *t*-test (with or without Bonferroni correction). Data are presented as the mean ± standard deviation. P-values < 0.05 were considered statistically significant.

## Supplementary information

Supplementary Figures 1 - 6

## Data Availability

The datasets generated during the current study are available from the corresponding author on reasonable request.
