# Supplementary material for: Cathepsin B overexpression induces degradation of perilipin 1 to cause lipid metabolism dysfunction in adipocytes
Source: Sci Rep. 2020 Jan 20;10:634. doi: 10.1038/s41598-020-57428-6 (PMC6971249; doi:10.1038/s41598-020-57428-6)
Supplement: Supplementary file 1 — Supplementary Figures 1 - 6 [file 41598_2020_57428_MOESM1_ESM.pdf]

Supplementary Information

Title

Cathepsin B overexpression induces degradation of perilipin 1 to cause lipid metabolism dysfunction in adipocytes

Author names and affiliations

Yuhei Mizunoe,[1†] Masaki Kobayashi,[2†] Shunsuke Hoshino,[2†] Ryoma Tagawa,[2] Rei Itagawa,[2] Ayana Hoshino,[2] Naoyuki Okita,[3] Yuka Sudo,[2] Yoshimi Nakagawa,[1,4] Hitoshi Shimano,[1,4,5,6] and Yoshikazu Higami[2*]

[1]Department of Internal Medicine (Endocrinology and Metabolism), Faculty of Medicine, University of Tsukuba, Ibaraki, Japan

[2]Laboratory of Molecular Pathology & Metabolic Disease, Faculty of Pharmaceutical Sciences, Tokyo University of Science, Chiba, Japan,

[3]Department of Pathological Biochemistry, Faculty of Pharmaceutical Sciences, Yamaguchi Tokyo University of Science, Yamaguchi, Japan,

[4]International Institute for Integrative Sleep Medicine (WPI-IIIS), University of Tsukuba, Tsukuba, Japan.

[5]Life Science Center for Survival Dynamics, Tsukuba Advanced Research Alliance (TARA), University of Tsukuba, Ibaraki, Japan.

[6]AMED-CREST, Japan Agency for Medical Research and Development (AMED), Tokyo, Japan.

†These authors contributed equally to this work.

*Corresponding author

Yoshikazu Higami, M.D., Ph.D., Laboratory of Molecular Pathology & Metabolic Disease, Faculty of Pharmaceutical Sciences, Tokyo University of Science, 2641 Yamazaki, Noda, Chiba 278-8510, Japan

Tel/Fax: +81-4-7121-3676; E-mail: higami@rs.noda.tus.ac.jp

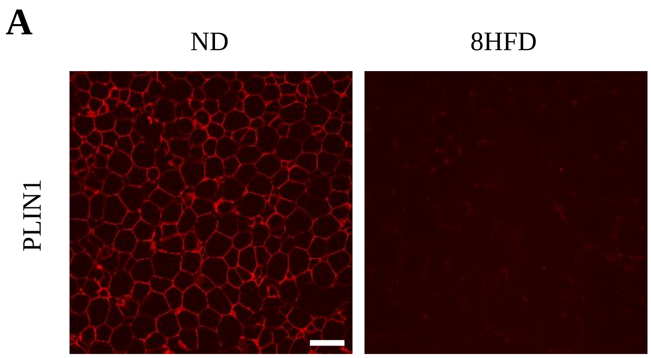

Supplementary Fig. 1. Downregulation of PLIN1 and upregulation of CTSB expression in obese WAT. (A) Immunofluorescent staining of PLIN1 (anti-PLIN1, #3470, 1:1000, Cell Signaling Technology) in WAT from ND and 8HFD mice. Scale bars represent 200 μm.

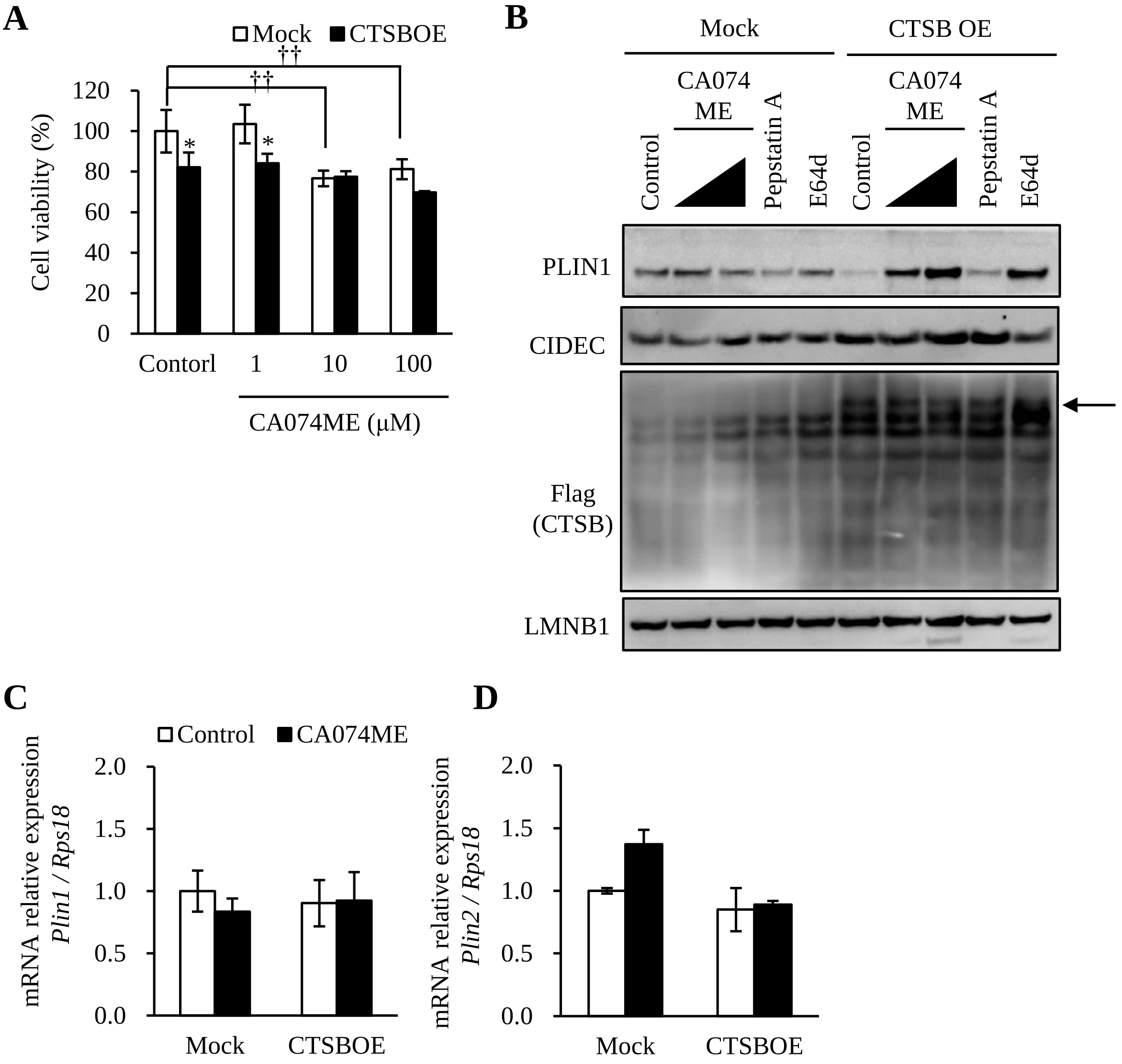

Supplementary Fig. 2. The effect of CA074ME, a CTSB inhibitor, on CTSB-OE 3T3L1 adipocytes. (A) The cytotoxic activity of CA074ME (1, 10 and 100 μM) against Mock or CTSB-OE 3T3L1 adipocytes was analyzed by WST assay. The data are represented as relative cell viabilities compared with Mock/3T3L1 adipocytes cultured without CA074ME (Control). (B) Mock and CTSB-OE 3T3L1 adipocytes were treated with Control (DMSO), 1 or 10 μM CA074ME, 10 μg/ml pepstatin A and 10 μg/ml E64d for 24 h, then harvested. Total cell lysates were analyzed by immunoblotting using anti-PLIN1, CIDEC, Flag, and LMNB1 antibodies. (C and D) qRT-PCR analysis for *Plin1* and *Plin2* mRNA levels in Mock or CTSB-OE 3T3L1 adipocytes. Values indicate the mean ± SD. Differences between values were analyzed by the Tukey-Kramer method. *P < 0.05, **P < 0.01 (n = 4).

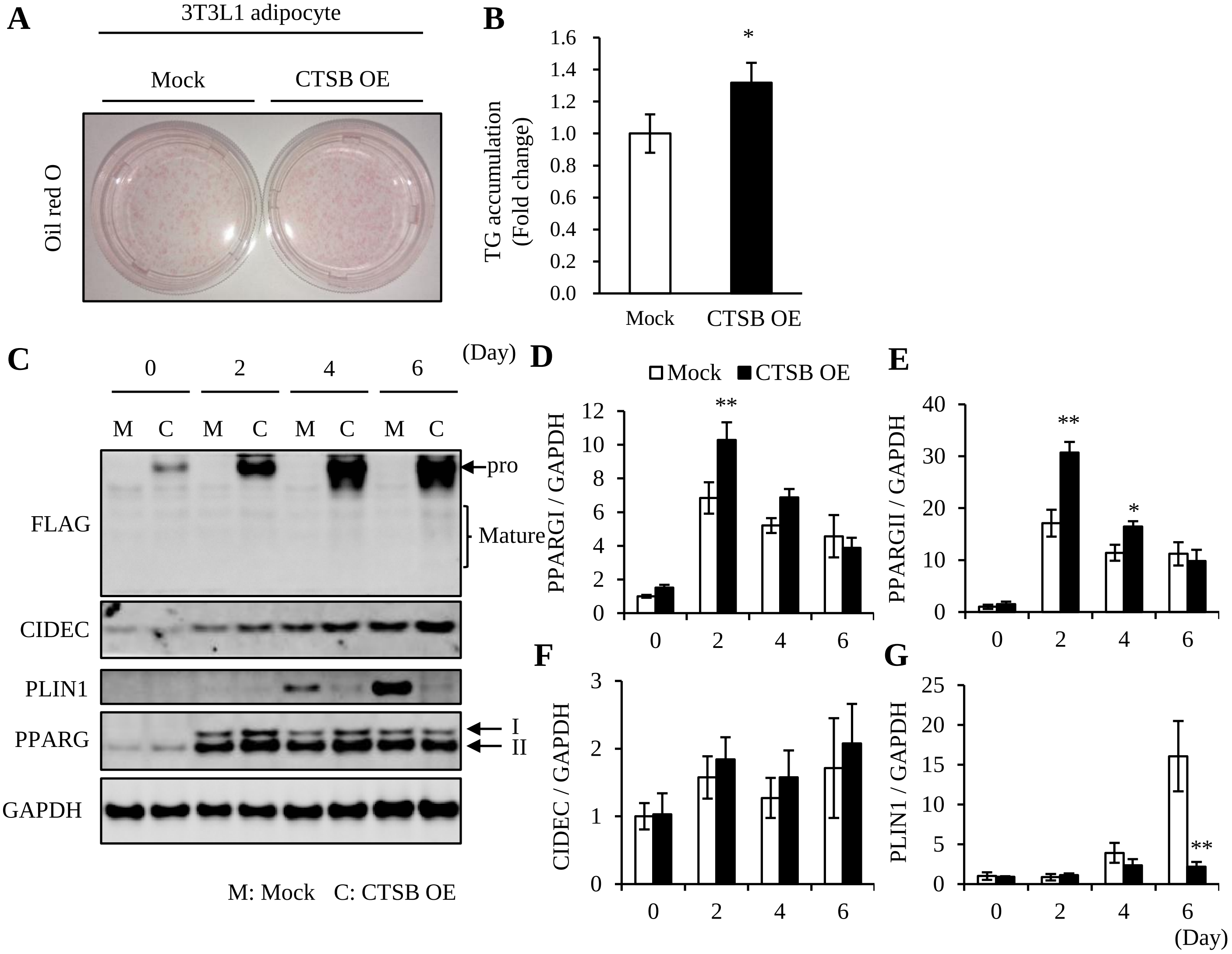

Supplementary Fig. 3. Upregulation of adipocyte differentiation by CTSB overexpression. (A and B) Mock or CTSB-OE 3T3L1 adipocytes were stained with Oil Red O. A representative image (A) and quantitative data (B) are shown. Values indicate the mean ± SD. Differences between values were analyzed by Student's *t*-test. *P < 0.05, **P < 0.01. (C-G) Mock and CTSB-OE 3T3L1 adipocytes were harvested at the indicated time points. Total cell lysates were analyzed by immunoblotting using anti-PLIN1, CIDEC, FLAG, PPARG, and LMNB1 antibodies (C) and quantified (D-G). Representative images and quantitative data (n = 4) are shown. Intensity of LMNB1 was used as a loading control. Values indicate the mean ± SD. Differences between values were analyzed by the Tukey-Kramer method. *P < 0.05, **P < 0.01.

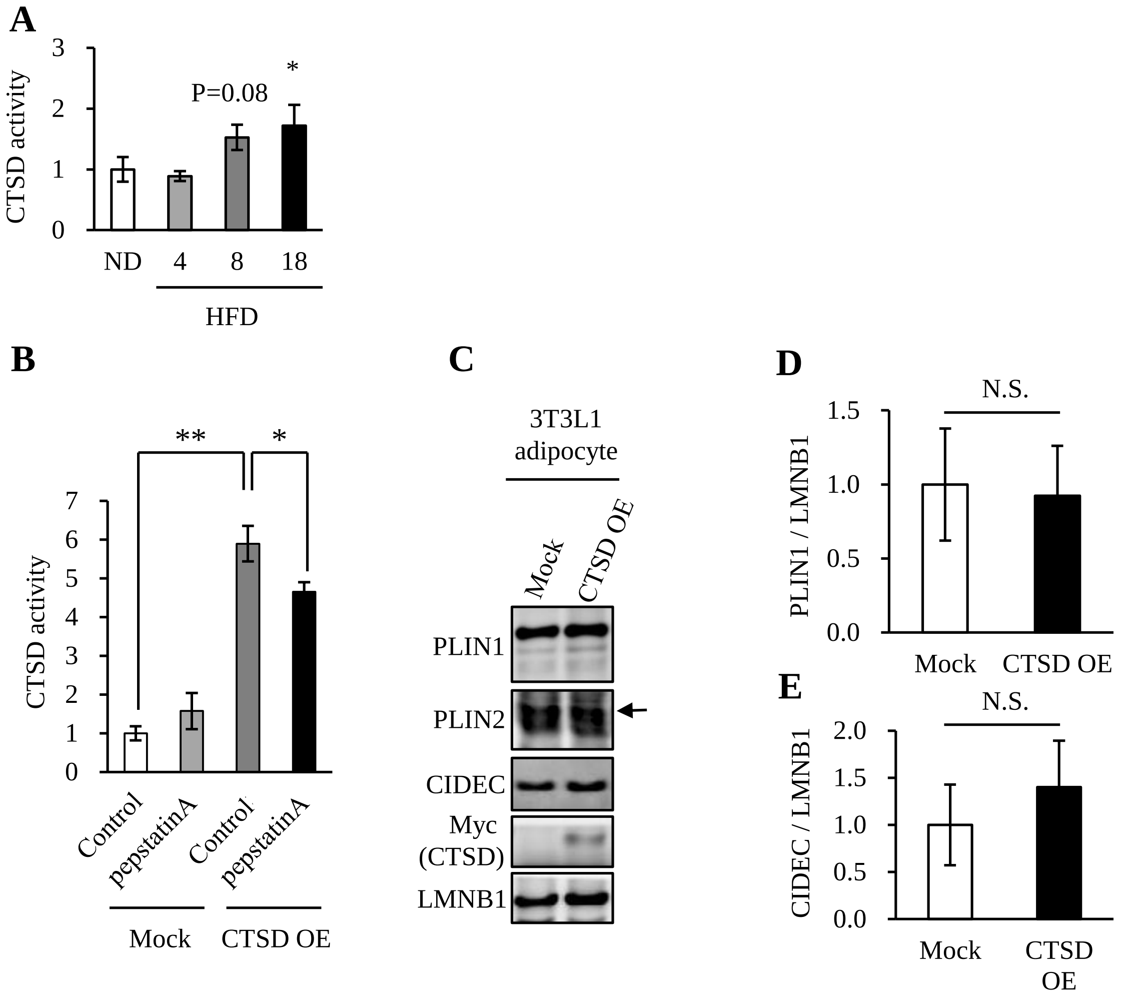

Supplementary Fig. 4. Cathepsin D (CTSD) overexpression does not affect the expression of PLIN1 protein. (A) Enzymatic assay of CTSD in the WAT of ND, 4HFD, 8HFD, and 18HFD mice, as analyzed by selective substrate. Values indicate the mean ± SD. Differences between values were analyzed by Student's *t*-test with Bonferroni correction. *P < 0.05, **P < 0.01. (B) Mock or CTSD-OE 3T3L1 adipocytes were treated with 10 μg/mL pepstatin A for 24 h. Enzymatic assays of CTSD were analyzed by selective substrate. Values indicate the mean ± SD. Differences between values were analyzed by the Tukey-Kramer method. *P < 0.05, **P < 0.01. (C–E) Total cell lysates were analyzed by immunoblotting using anti-PLIN1, PLIN2, CIDEC, MYC, and LMNB1 antibodies (C) and quantified (D and E). Representative images and quantitative data (n = 4) are shown. Intensity of LMNB1 was used as a loading control. Values indicate the mean ± SD. Differences between values were analyzed by Student's *t*-test. *P < 0.05, **P < 0.01.

A

Primer list for Retrovirus vector construction

CTSB	5′-GGG CTC GAG CAC CAT GTG GCA GCT CTG GGC C-3′(*Xho1*)<br>5′-GCC GCG GCC GCT TA<u>C TTA TCG TCA TCC TTG TAA TC</u>G ATC TTT TCC CAG TAC TGA TCG GTG-3′ (*Not1*)<br>with restriction enzyme sites indicated in italics and antisense of FLAG-tag coding sequences underlined
CTSD	5′-GGC CTC GAG CAC CAT GAA GAC TCC CGG CGT CTT GCT-3′(Xho1)<br>5′-GCC GCG GCC GCT TA<u>C AGA TCC TCT TCT GAG ATG AGT TTT TGT TC</u>G AGT ACG ACA GCA TTG GCA AAG C-3′(Not1)<br>with restriction enzyme sites indicated in italics and antisense of Myc-tag coding sequences underlined

B

Primer list for Quantitative real-time RT-PCR

Plin1	(forward) 5′-TGG GAA GCA TCG AGA AGG TG-3′
	(reverse) 5′-ATG GTG TGT CGA GAA AGA GTG TTG-3′
Plin2	(forward) 5′- CAG CCA ACG TCC GAG ATT G-3′
	(reverse) 5′-CAC ATC CTT CGC CCC AGT T-3′
Tbp	(forward) 5′-CAG TAC AGC AAT CAA CAT CTC AGC-3′
	(reverse) 5′-CAA GTT TAC AGC CAA GAT TCA CG-3′

Supplementary Fig. 5. Primer list for Retrovirus vector construction (A) and Quantitative real-time RT-PCR (B)

Figure. 1A

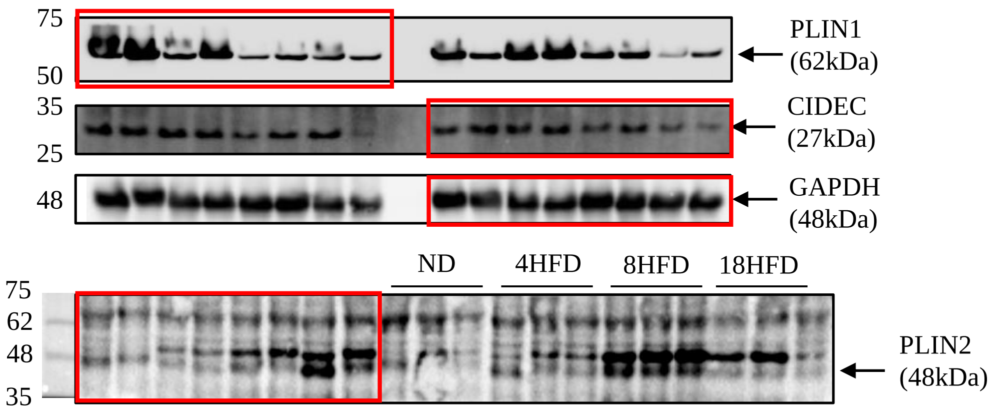

Figure. 2A

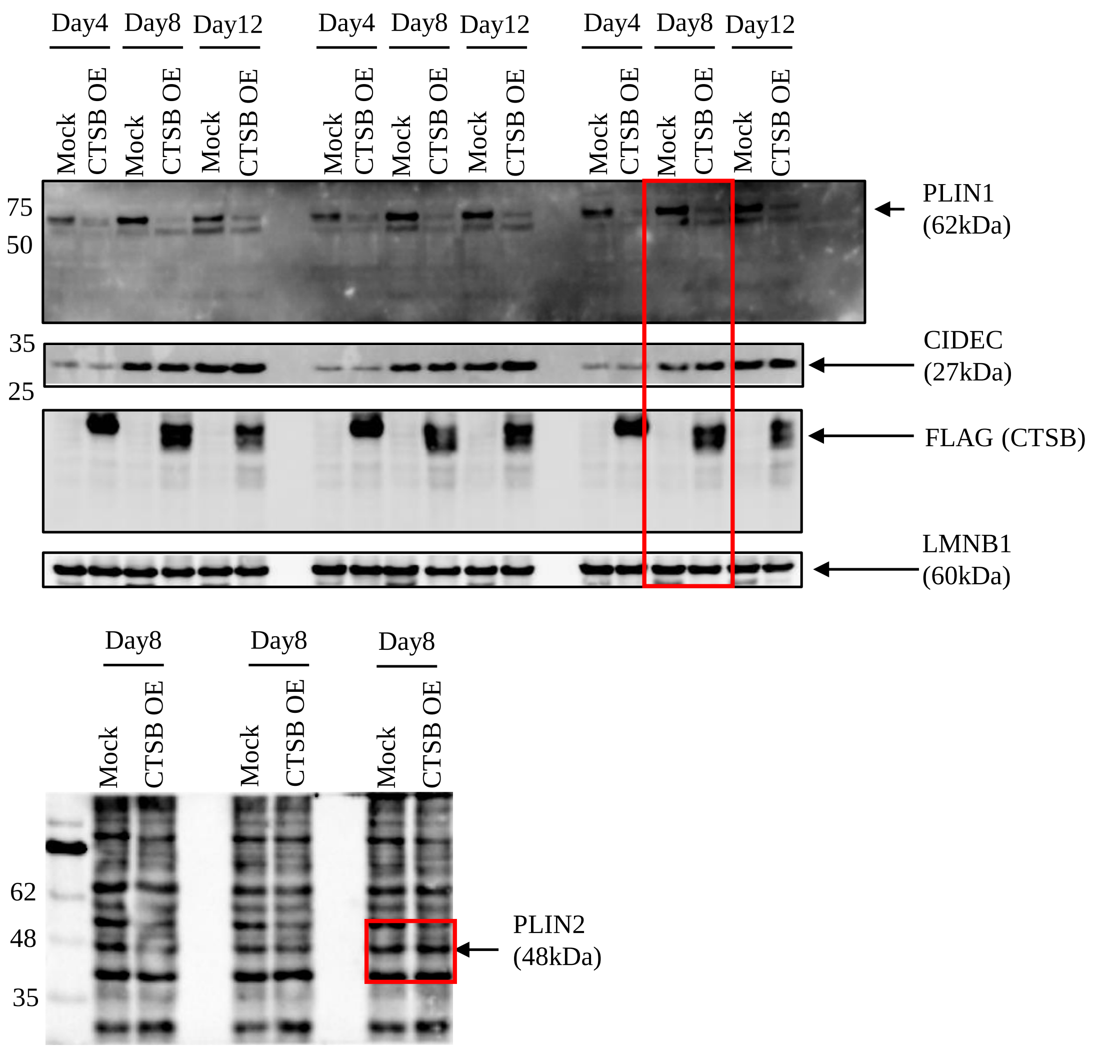

Supplementary Fig. 6. Full scans of western blots. Specific bands shown in the figures are highlighted by red boxes.
